# Supramolecular porphyrin as an improved photocatalyst for chloroform decomposition

**DOI:** 10.1039/d2ra07720e

**Published:** 2023-02-13

**Authors:** J. M. S. Lopes, A. A. Batista, P. T. Araujo, N. M. Barbosa Neto

**Affiliations:** a Department of Physics, Federal University of Roraima Boa Vista RR Brazil lopesjefferson01@yahoo.com.br; b Department of Chemistry, Federal University of São Carlos São Carlos SP Brazil; c Department of Physics and Astronomy, University of Alabama Tuscaloosa Alabama USA paulo.t.araujo@ua.edu; d Institute of Natural Sciences, Graduate Program in Physics, Federal University of Pará Belém PA Brazil barbosaneto@ufpa.br

## Abstract

In this work, the outlying decoration of the free-base *meso*-(4-tetra) pyridyl porphyrin (H_2_TPyP) with the RuCl(dppb)(5,5′-Me-bipy) ruthenium complex (here named Supra-H_2_TPyP) is observed as an improved molecular photocatalyst for dye-mediated chloroform (CHCl_3_) decomposition *via* one-photon absorption operating in the visible spectral range (532 nm and 645 nm). Supra-H_2_TPyP offers a better option for CHCl_3_ photodecomposition when compared to the same process mediated by pristine H_2_TPyP, which requires either excited-state- or UV absorption. The chloroform photodecomposition rates for Supra-H_2_TPyP as well as its excitation mechanisms are explored as a function of distinct laser irradiation conditions.

## Abstract 2

The photocatalytic ability of free base pyridyl porphyrin to decompose chloroform is improved when ruthenium complexes are attached to its structure, enabling the use of lower energy one-photon excitations.

## Graphical abstract

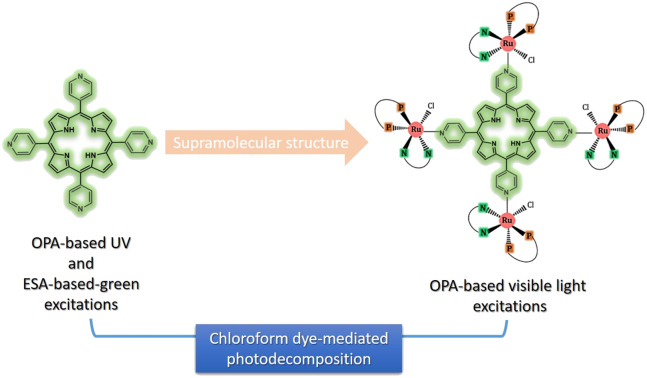

## 1. Introduction

Chlorine-derived disinfectants like sodium hypochlorite, chloramine, and chlorine dioxide are important structures for the treatment of drinking water and swimming pools.^1–5^ Despite their good effectiveness in killing pathogenic microorganisms, unfortunately, when these substances interact with natural organic matter (*e.g.*, urine, sweat, and saliva) non-negligible amounts of undesired byproducts can be generated. Among them, chloroform (CHCl_3_) brings significant concerns to health due to its cytotoxic effects.^1,6–8^ In recent years, two major efforts have been conducted to deal with this problem: (1) the development of novel molecular sensors to detect chloroform in water,^9–11^ and (2) the creation of methodologies to decompose this molecule in solution. Concerning the effort (2), although the biotechnological^12,13^ and the pulsed-corona-discharges-assisted^14^ approaches have been lately tested, the dye-catalyzed photochemical-based approaches stand as low-cost, non-invasive, selective, and controllable alternatives.^15–20^

The excited-state dynamics exhibited by porphyrins^21–25^ set them as versatile molecular photocatalysts^15,26–29^ that have been explored in fields like dye-sensitized solar cells,^30–33^ photomedicine,^34–36^ and artificial photosynthesis.^37–39^ In the last decades, these macrocycle molecules have been explored as photocatalysts for dye-mediated decomposition of chloroform (CHCl_3_) under one-photon absorption (OPA) and/or excited-state absorption (ESA).^15,16^ Differently from visible light OPA, ultra-violet OPA (*λ*_exc_ = 266 nm) accesses the porphyrin's photooxidative excited states enabling the decomposition of CHCl_3_ through an oxi-reduction reaction, which yields hydrochloric acid (HCl) and other products.^15^ More recently, ESA^16^ became an alternative approach to employ visible radiation for CHCl_3_ photodecomposition.

The tunning of the excitation energy threshold for photooxidation could allow the photo-reaction to occur at lower- (higher-) energy OPA (ESA) but is still poorly explored. The synthesis of novel porphyrinic structures could facilitate such energy threshold controllability. Examples of synthetic approaches are: (1) the inner and outer macrocycle decoration;^21,40,41^ (2) oligomer formation involving one or more types of porphyrins;^38,42–44^ and (3) supramolecular porphyrin structures.^26,45–51^ The attachment of metallic complexes (*e.g.*, ruthenium-, rhenium- and iridium-derived) at the outer positions of porphyrins generates supramolecular structures that offer novel excited states for achieving the photo-reaction threshold while keeping original states from both the pristine porphyrin and the complexes.^46,48,49,52–56^

In this work, a supramolecular structure originated from the attachment of the RuCl(dppb)(5,5′-Me-bipy) ruthenium complex at each (4-pyridyl) site of the *meso*-tetra(4-pyridyl) free-base porphyrin (H_2_TPyP) is used as a molecular photocatalyst for CHCl_3_ decomposition, allowing the reaction to occur *via* visible-light OPA. The influence of the excitation wavelength and mechanisms on the photo-reaction rates is also explored.

## 2. Materials and methods

### 2.1 Samples and spectroscopic measurements

**Fig. 1:**
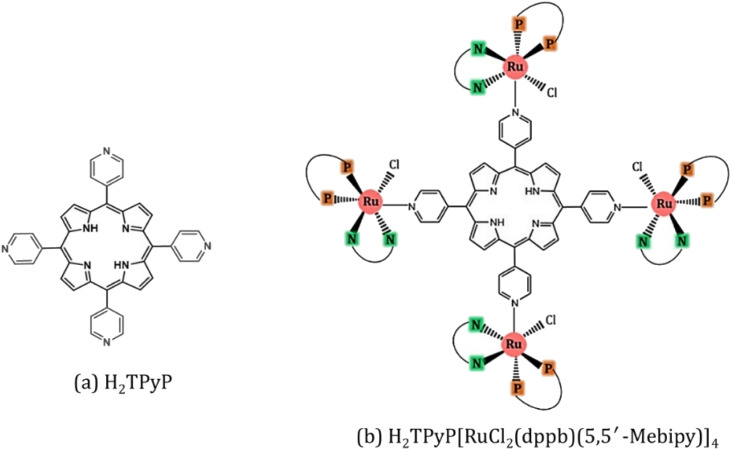
(a) H_2_TPyP and (b) Supra-H_2_TPyP, with RuCl_2_(dppb)(5,5′-Mebipy) ruthenium complexes attached at each (4-pyridyl) site of H_2_TPyP. Here, 5,5′-Mebipy stands for 5,5′-dimethyl-2,2′-bipyridine (N–N), and dppb stands for 1,4-bis(diphenylphosphine) butane (P–P).

The synthesis of H_2_TPyP and its supramolecular H_2_TPyP[RuCl(dppb)(5,5′-Me-bipy)]_4_ structure (Supra-H_2_TPyP, see Fig. 1) follows the procedures described in the literature.^47,57^ The spectroscopic measurements were conducted with samples dissolved in CHCl_3_ stabilized with amylene (NEON Inc.) used as received. Solution concentrations were kept below 10.0 μM to prevent spontaneous aggregation and inner filter effects. Absorption spectra were acquired with a JASCO V-670 spectrophotometer whereas steady-state photoluminescence (PL) spectra were measured using a setup composed of a Xenon lamp (ACTON); a monochromator (ACTON, model 300i) and a portable spectrophotometer (Ocean Optics), with the PL signal being detected in a 90° geometry relative to the excitation beam direction. Time-resolved PL experiments were conducted using a Time-Correlated Single Photon Counting (TCSPC) system (Horiba, Delta-Flex model with 27 ps of temporal resolution), equipped with a pulsed excitation source (*λ*_exc_ = 352 nm with 8.0 MHZ of repetition rate). The PL decays were collected at the maximum of each spectrum. Emission quantum yields^58,59^ were calculated adopting H_2_TPyP dissolved in CHCl_3_ as standard^22^ and applying eqn (1):1
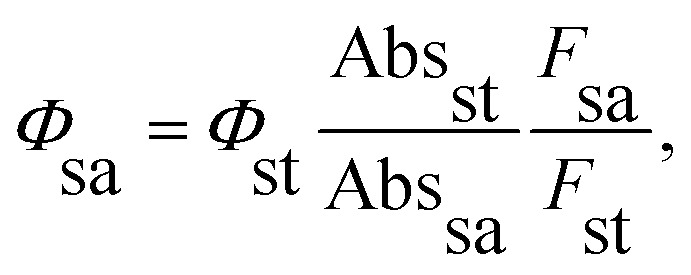
where, *Φ*_sa_ and *Φ*_st_ stand for the quantum yields of the sample (sa) and standard (st) solution, respectively. The quantities Abs_st_ (F_st_) and Abs_sa_ (*F*_sa_) are the absorbances at 420 nm (the integrated PL spectrum for the corresponding excitation) from the sample and standard solutions.

### 2.2. Photochemistry assays

For OPA in porphyrins, two sources (purchased from Laserline Inc), green-OPA (532 nm; ∼4.0 W cm^−2^, 19 200 J cm^−2^ total incident fluence, 90.0 minutes of irradiation) and red-OPA (635 nm; ∼5.0 W cm^−2^, 30 000 J cm^−2^ total incident fluence, 90.0 minutes of irradiation) were utilized. Here, ESA is promoted by a frequency-doubled Q-switched Nd-YAG laser (Quantel, model Q-smart 100, 6.0 ns FWHM, 532 nm excitation, 20.0 Hz of repetition rate, total incident fluence regime of ∼2500 J cm^−2^). The quantitative analysis of all irradiation conditions was performed by considering the effectively absorbed fluence instead of incident fluence. The relation between incident (*F*_I_), transmitted (*F*_T_) and absorbed (*F*_A_) fluences is written as *F*_I_ = *F*_T_ + *F*_A_, or 
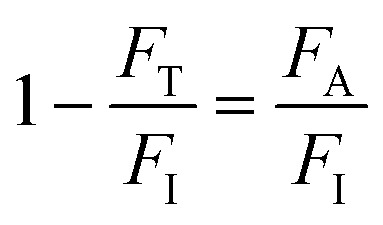
.^58^ From the Lambert–Beer law,^58^
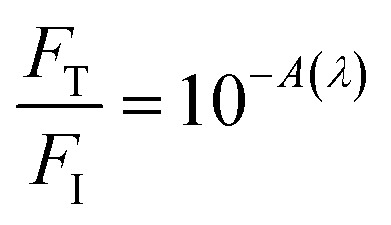
 (with *A*(*λ*) corresponding to absorbance at the incident wavelength, for the non-irradiated solution) resulting in eqn (2):

2 *F*_A_ = *F*_I_(1 − 10^−*A*(*λ*)^)

During all the spectroscopic measurements and irradiation processes, the samples were placed in sealed quartz cuvettes of 1.0 cm path length with four polished windows.

Control samples of H_2_TPyP and Supra-H_2_TPyP dissolved in CHCl_3_ were kept in a dark environment at room temperature (∼22.5 °C) to evaluate their stability. Absorption spectra confirm that no spontaneous modifications occur in both samples for 240.0 minutes of storage, endorsing their stability. This implies their durability is at least three times greater than the time demanded to perform the photo-induced reactions discussed herein.

## 3. Results and discussions

### 3.1 Photophysics of Supra-H_2_TPyP

**Fig. 2:**
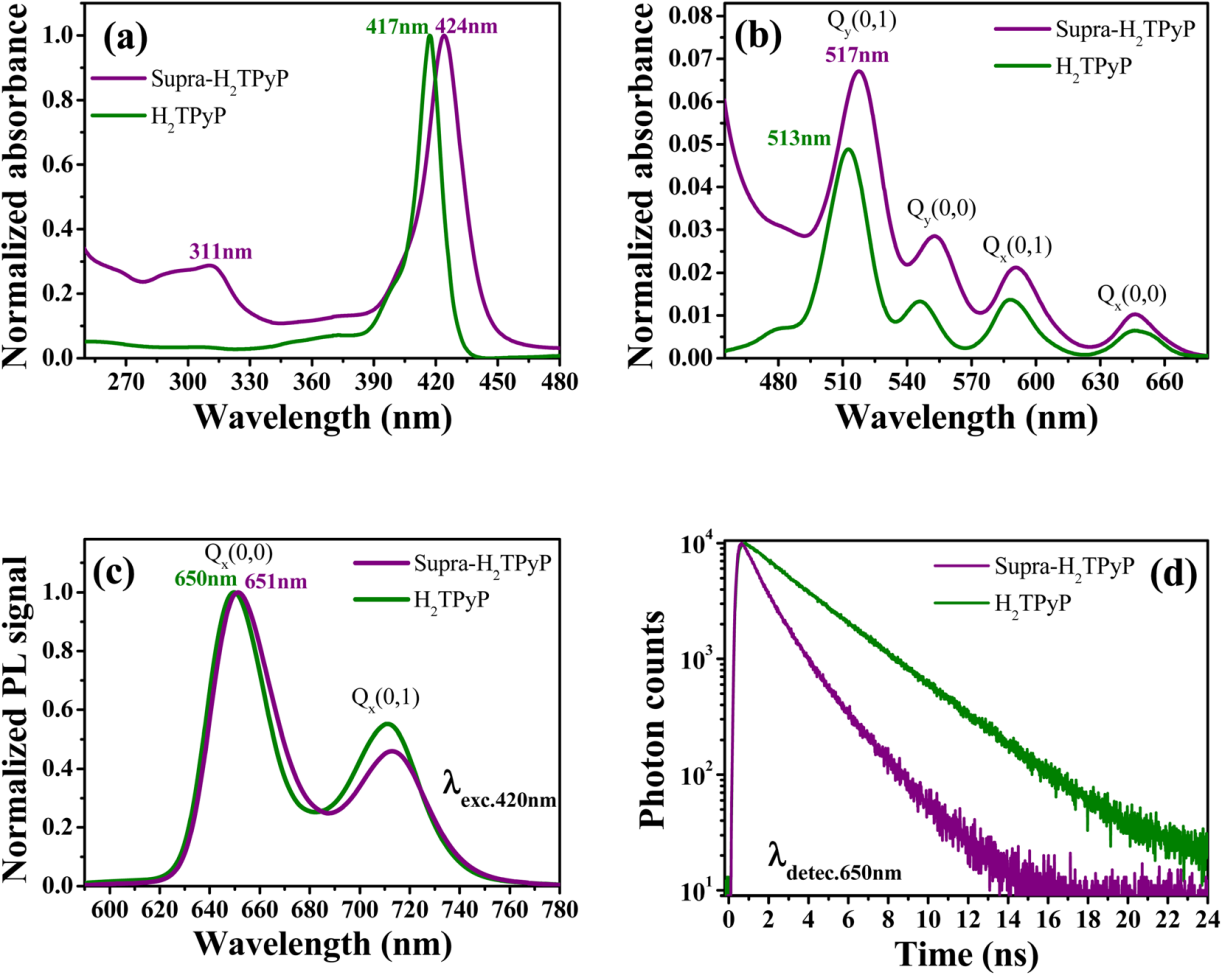
Normalized absorption spectrum at (a) B- and (b) Q-bands. (c) Normalized PL spectrum (*λ*_exc._ = 420 nm) and (d) fluorescence decays (*λ*_detec._ = 650 nm) for H_2_TPyP (green curves) and Supra-H_2_TPyP (purple curves).

As shown in Fig. 2, the absorption spectrum of Supra-H_2_TPyP displays red-shifted B- and Q-bands relative to H_2_TPyP, in agreement with literature.^16,60^ Additionally, signatures belonging to the metallic complex are also observed (see intraligand bands located at around 311 nm).^47,61–65^ Due to their superposition with H_2_TPyP bands, spectral signatures associated with the complex's MLCT bands centered around 470 nm are not resolved but lead to an overall increase of the spectrum baseline^49,52,56^ (Fig. 2b).

The Supra-H_2_TPyP PL spectrum (Fig. 2c) shows a small redshift (∼1 nm) regarding H_2_TPyP. This effect is accompanied by obtaining a 72% lower emission quantum yield for Supra-H_2_TPyP concerning H_2_TPyP^22^ (see Table 1), in agreement with previous reports.^49–52^ The Supra-H_2_TPyP also presents a variation in the relative intensity between electronic (Q(0,0)) and vibronic (Q(0,1)) emission bands with relation to H_2_TPyP, which indicates that either (1) the involved H_2_TPyP vibronic progressions are changing^52,53^ or (2) a new band arises and overlaps with H_2_TPyP original bands.^52,53^ From TCSPC experiments it is observed that the mono-exponential decay (7.26 ns) exhibited by H_2_TPyP in CHCl_3_^60^ evolves to a bi-exponential decay (*τ*_1_ = 2.01 ns (54%) and *τ*_2_ = 4.54 ns (46%)) in Supra-H_2_TPyP (Fig. 2d and Table 1). Based on the literature,^49,52,53^ and considering that isolated ruthenium complexes do not exhibit emission bands around the probed wavelength (650 nm), *τ*_1_ is associated with Supra-H_2_TPyP, while the lifetime *τ*_2_ is associated with the perturbed H_2_TPyP deactivation pathway.^49,52,53^ This trend supports the hypothesis of a novel band in the steady-state PL spectrum^53^ (Fig. 2c).

**Table 1:** Photophysical features of H_2_TPyP and Supra-H_2_TPyP, dissolved in CHCl_3_. Here, *τ*_2_ (*τ*_1_) refers to the excited state lifetime of the former (newly-formed) relaxation pathway

	*B*_max_ (nm)	*Q*_max_ (nm)	PL_max_ (nm)	*τ*_1_ (ns)	*τ*_2_ (ns)	*Φ* (× 10^−2^)
H_2_TPyP	417	513	650	—	7.26 (100%)	1.61
Supra-H_2_TPyP	424	517	651	2.01 (54%)	4.54 (46%)	0.45

### 3.2 Green-OPA irradiation of Supra-H_2_TPyP

**Fig. 3:**
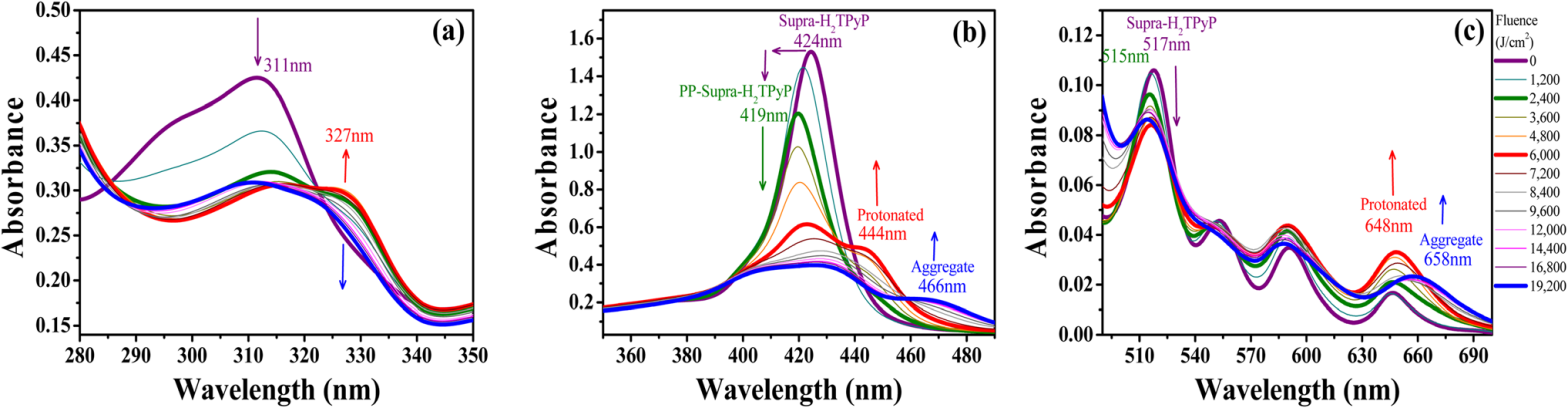
Evolution of the absorption spectrum of Supra-H_2_TPyP under green-OPA excitation (532 nm, 0–19200 J cm^−2^) at (a) the UV bands (from 280 nm to 350 nm), (b) the B-band, and (c) the Q-band.

In chloroform, H_2_TPyP is photostable under green-OPA irradiation.^15,16^ This is not true for Supra-H_2_TPyP under the same irradiation condition. Fig. 3 shows that the spectroscopic signatures of Supra-H_2_TPyP are significantly modified as a function of the incident fluence. Because the modifications occur in different stages, the results are separately discussed in three fluence ranges: (i) 0–2400 J cm^2^; (ii) 2400–6000 J cm^2^; and (iii) 6000–19 200 J cm^−2^.

In range (i), both B- and Q-bands undergo a blue shift. The main Supra-H_2_TPyP B-band, originally centered at 424 nm (0 J cm^−2^) (purple solid line), downshifts to 419 nm at 2400 J cm^−2^ (olive solid line) while the Q_*y*_(0,1) downshifts from 517 nm to 515 nm. This behavior suggests that Supra-H_2_TPyP is undergoing a similar photodissociation as reported for the H_2_TPyP[RuCl_2_(CO)(PPh_3_)_2_]_4_ tetraruthenated porphyrin.^53^ The occurrence of photodissociation in the fluence range (i) would imply: (1) the decomposition of ruthenium complexes; and (2) the absence of processes associated with the fluence ranges (ii) and (iii).^15,16^ However, our data show that (1) and (2) do not happen, see Fig. 3. It is seen that in the fluence range (i), the RuCl(dppb)(5,5′-Me-bipy) complexes in Supra-H_2_TPyP, with intraligand bands spanning from ∼280–360 nm, undergo a sort of transformation, forming a novel supramolecular species, baptized PP-Supra-H_2_TPyP. The determination of the type of transformation that is photomodifying RuCl(dppb)(5,5′-Me-bipy) is out of the scope of this work but we understand that photooxidation,^66,67^ photoisomerization,^68–70^ and photoinduced ligand loss^71,72^ are possible processes driving the phenomenon.

In range (ii), the peak at 419 nm no longer blueshifts and starts developing into a novel spectroscopic signature centered at 444 nm, see Fig. 3b. The rise of this new signature occurs simultaneously with the enhancement of the Q_*x*_(0,0)-band (around 648 nm). These are signatures associated with photo-protonation of PP-Supra-H_2_TPyP.^15,16,73–75^ Moreover, the PP-Supra-H_2_TPyP 419 nm peak redshifts in ≈2 nm, which is a signature for the protonation of the photomodified RuCl(dppb)(5,5′-Me-bipy).^16,76^ In fact, a closer inspection of RuCl(dppb)(5,5′-Me-bipy) intraligand bands shows that the peak at ∼327 nm is enhanced (Fig. 3a). It is important to recall that protonation requires acid environments. This is reported for complexes similar to RuCl(dppb)(5,5′-Me-bipy), in which an acidic solution was created through the addition of aliquots of HCl.^77,78^ Since we do not add any acid to our solutions, the observation of protonation of both the porphyrin and the photomodified RuCl(dppb)(5,5′-Me-bipy) confirms the release of HCl in the solution as a consequence of CHCl_3_ photodecomposition *via*green-OPA.

In the range (iii), it is observed that protonated PP-Supra-H_2_TPyP signatures, B-(≈444 nm) and Q_*x*_(0,0)-(≈648 nm) bands, disappear, which is followed by the rise of new bands at ≈466 nm and ≈658 nm, respectively. The new features indicate that the protonated PP-Supra-H_2_TPyP is forming aggregates (possibly J-aggregates), similar to those previously reported for H_2_TPyP under ESA excitation.^16^ It is also observed that the intraligand band of the photomodified RuCl(dppb)(5,5′-Me-bipy) at 327 nm undergoes a decrease in intensity with no indications of further transformations.

**Fig. 4:**
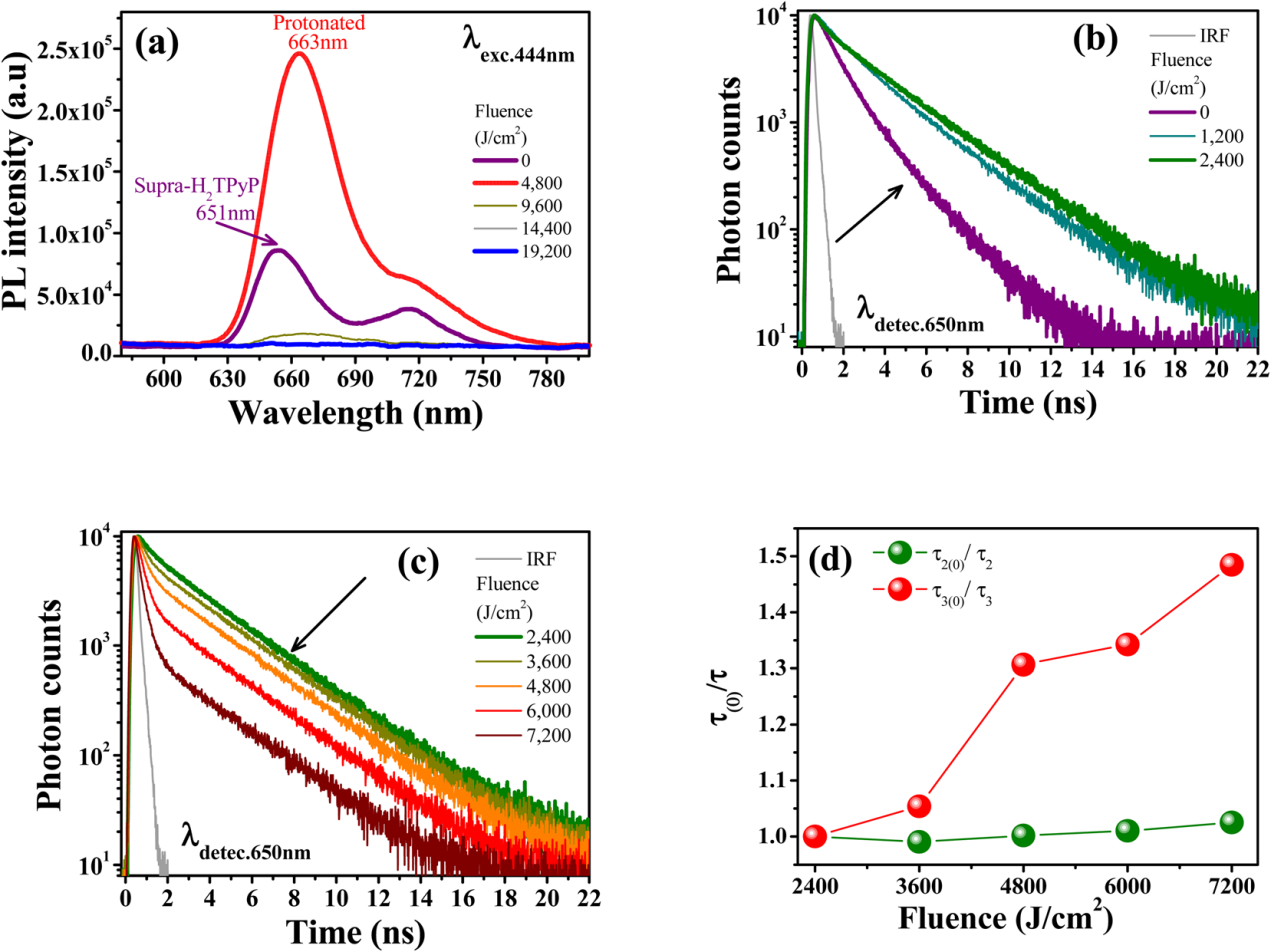
(a) PL spectra evolution with increasing fluence (*λ*_exc._ = 444 nm). Supra-H_2_TPyP PL decay (*λ*_detec._ = 650 nm) under green-OPA (*λ*_exc._ = 532 nm) for: (b) fluence range (i), and (c) fluence range (ii). (d) Fluence dependence of the *τ*_(0)_/*τ* ratio for PP-Supra-H_2_TPyP (green spheres) and its protonated counterpart (red spheres).

The protonation of Supra-H_2_TPyP also induces new signatures in the steady-state PL spectrum, which are in agreement with the results discussed above for UV-Vis absorption. Fig. 4a shows that the Supra-H_2_TPyP PL spectrum (purple solid line) evolves to the protonated PL spectrum (red solid line).^16^ After protonation, the PL signal continuously decreases with increasing fluence, becoming almost null at 19 200 J cm^−2^. This decrease in the PL magnitude is associated with a reduction in the emission quantum yield of the protonated species, which is characteristic of J-aggregation in range (iii).^16,74,79–83^

Fig. 4b shows that the Supra-H_2_TPyP PL decay at 0 J cm^−2^ (purple solid line), which initially displays two characteristic lifetimes (4.54 ns (46%) and 2.01 ns (54%)), continuously evolves into the PP-Supra-H_2_TPyP decay profile at 2400 J cm^−2^, with a dominant characteristic lifetime of ≈7.01 ns (91%). A minor contributing lifetime of ≈ 3.17 ns (4%) is also measured and understood to belong to the remaining Supra-H_2_TPyP. In addition, a new PL decay with a characteristic lifetime of 0.98 ns (5%) associated with the protonated PP-Supra-H_2_TPyP is observed, see Table 2. The results in Fig. 4c and Table 2 support that in the range (i) both Supra-H_2_TPyP and PP-Supra-H_2_TPyP coexist. When the range (ii) starts (2400 J cm^−2^), PP-Supra-H_2_TPyP becomes predominant, coexisting with both Supra-H_2_TPyP and the protonated PP-Supra-H_2_TPyP. From 3600 J cm^−2^ on, the percentage contribution of the lifetime associated with Supra-H_2_TPyP becomes negligible.

**Table 2:** Excited state decay parameters showing the evolution of Supra-H_2_TPyP under green-OPA. The values in parentheses in the lifetime columns represent the percentage contribution of each fitting exponential. *χ*^2^ estimates the fitting precision

Fluence (J cm^−2^)	*τ*_1_ (ns)	*τ*_2_ (ns)	*τ*_3_ (ns)	*χ* ^2^
0	2.01 (54%)	4.54 (46%)	—	1.06
1200	2.16 (17%)	6.39 (83%)	—	1.03
2400	3.17 (4%)	7.01 (91%)	0.98 (5%)	1.07
3600		7.08 (89%)	0.93 (11%)	1.04
4800		7.00 (82%)	0.75 (18%)	1.06
6000		6.94 (65%)	0.73 (35%)	1.02
7200		6.84 (42%)	0.66 (58%)	1.12

At 7200 J cm^−2^, the PP-Supra-H_2_TPyP (protonated PP-Supra-H_2_TPyP) lifetime decreases from 7.01 ns (0.98 ns) to 6.84 ns (0.66 ns). Fig. 4d shows that, at 7200 J cm^−2^, the quenching of these lifetimes, *τ*_2(0)_/*τ*_2_ (PP-Supra-H_2_TPyP) and *τ*_3(0)_/*τ*_3_ (protonated PP-Supra-H_2_TPyP), are 1.02 and 1.47, which originates from exciplex formation.^22^ The quenching of the protonated PP-Supra-H_2_TPyP is 144% more effective in comparison with that for PP-Supra-H_2_TPyP. This result supports the presence of electrostatically-enhanced exciplex formed between the positively charged protonated PP-Supra-H_2_TPyP and Cl^−^ ions dispersed in the solution.^16^ No discussion is addressed for range (iii) because the decrease in PL intensity invisibilized the PL decay measurements.

As discussed in the literature,^15,16^ free base porphyrins (H_2_TPyP and H_2_TPP) under UV-OPA (*λ*_exc_ = 266 nm) and ESA (*λ*_exc_ = 532 nm) excitations are able to photodecompose CHCl_3_ and form HCl with excitation energy thresholds located above the B-band energy (≈2.97 eV).^15,16^ This implies that these free-base porphyrins never underwent photoprotonation under OPA operating at 475 nm (2.61 eV) or 532 nm (2.33 eV).^15,16^ In this work, we were able to photoprotonate PP-Supra-H_2_TPyP dissolved in CHCl_3_ under green-OPA (≈2.33 eV), meaning HCl is being formed as a consequence of CHCl_3_ decomposition. This process should not happen if the ground and the excited states of PP-Supra-H_2_TPyP were similar in energy to the ground and the excited states of H_2_TPyP. Since the excitation gap is fixed by the CW laser energy employed in green-OPA (≈2.33 eV), our results indicate two possible scenarios: (1) the involved ground state in PP-Supra-H_2_TPyP must be higher in energy in comparison to the correspondent state in H_2_TPyP and/or (2) the photooxidative excited state in PP-Supra-H_2_TPyP must be lower in energy concerning H_2_TPyP.

### 3.3 Other irradiation conditions

**Fig. 5:**
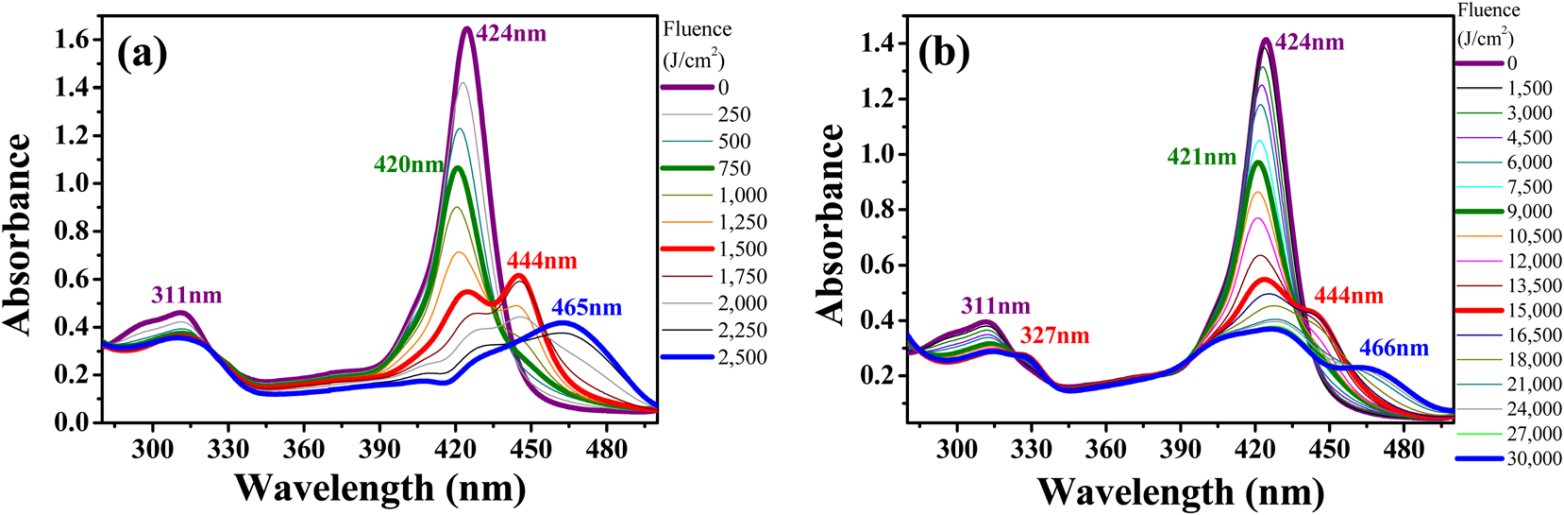
Evolution of the absorption spectrum of Supra-H_2_TPyP dissolved in CHCl_3_ and irradiated under (a) green-ESA (532 nm, 6 ns, 0–2500 J cm^−2^) and (b) red-OPA (633 nm, 0–19 200 J cm^−2^).

Fig. 5a and b show the same photo-induced modifications (PP-Supra-H_2_TPyP formation, photo-protonation, and photo-aggregation) in the spectroscopic signatures of Supra-H_2_TPyP for green-ESA and red-OPA irradiations, respectively. The successful decomposition of CHCl_3_ under red-OPA irradiation (photon energy delivered ≈1.96 eV) sets a new lower limit for the excitation energy required to trigger the process.

**Fig. 6:**
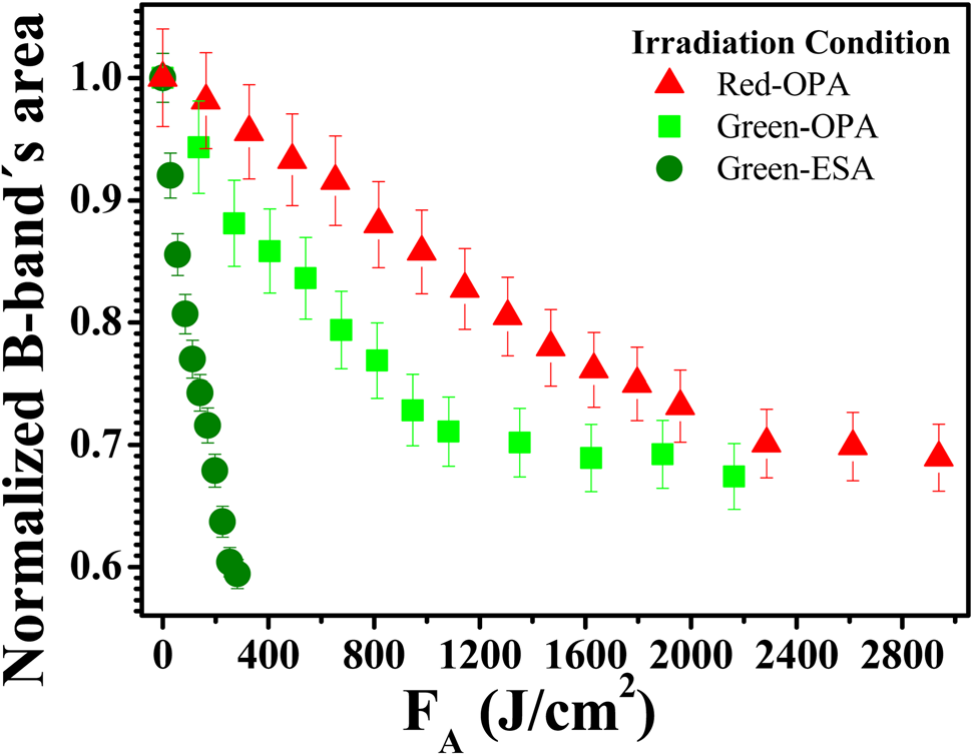
Absorbed fluence-dependent evolutions of normalized B band's area comparing green-ESA (filled olive spheres), green-OPA (filled light-green circles), and red-OPA (filled red triangles) excitations applied to Supra-H_2_TPyP.

To understand the photodecomposition rates under the different laser irradiation conditions, the Supra-H_2_TPyP B-band integrated area (from 360 to 490 nm) for each laser absorbed fluence (*F*_A_) was normalized by the B-band integrated area (also from 360 to 490 nm) of the reference non-irradiated Supra-H_2_TPyP, and plotted as a function of *F*_A_ (see Fig. 6). The exponential profiles obtained under each laser irradiation condition were fitted using *A*_F_ = e^(−*kF*_A_)^, where *A*_F_ and *k* represent, respectively, the aforementioned normalized areas for each *F*_A_ and the net photomodification rate (given in cm^2^ J^−1^). The fitting results show that the rates obey the following hierarchy: green-ESA (*k* = 4.32 × 10^−3^ cm^2^ J^−1^) > green-OPA (*k* = 1.43 × 10^−3^ cm^2^ J^−1^) > red-OPA (*k* = 0.50 × 10^−3^ cm^2^ J^−1^), which shows that green-ESA leads to more efficient photoreaction processes in comparison with the OPA-based irradiations.

## 4. Conclusions

Supra-H_2_TPyP places itself as an excellent candidate for a broad-range OPA-visible-light active molecular photocatalyst in dye-mediated chloroform decomposition. This supramolecular structure displays good photoreaction rates under visible OPA irradiation, which is a simpler and more affordable excitation mechanism than visible ESA and UV-OPA. Our results show that Supra-H_2_TPyP when combined with different excitation wavelengths can be used to controllably photodecompose CHCl_3_ and, consequently, controllably photoprotonate Supra-H_2_TPyP itself, which is also interesting for sensing chloroform in solution and other applications in fields like materials science.

## Author contributions

J. M. S. Lopes: data curation, writing-original draft, writing – review & editing, investigation, visualization, formal analysis, methodology. A. A. Batista: writing-review & editing, data curation, formal Analysis, project administration, resources, validation. P. T. Araujo: data curation, writing-original draft, writing – review & editing, visualization, investigation, fund acquisition, supervision, project administration, validation, formal analysis, methodology. N. M. Barbosa Neto: data curation, writing – original draft, writing – review & editing, visualization, investigation, validation, formal analysis, methodology, supervision, resources, funding acquisition, project administration.

## Conflicts of interest

The authors declare that they have no known competing financial interests or personal relationships that could have appeared to influence the work reported in this manuscript.

## Supplementary Material
